# Levosimendan for resuscitating the microcirculation in patients with septic shock: a randomized controlled study

**DOI:** 10.1186/cc9387

**Published:** 2010-12-23

**Authors:** Andrea Morelli, Abele Donati, Christian Ertmer, Sebastian Rehberg, Matthias Lange, Alessandra Orecchioni, Valeria Cecchini, Giovanni Landoni, Paolo Pelaia, Paolo Pietropaoli, Hugo Van Aken, Jean-Louis Teboul, Can Ince, Martin Westphal

**Affiliations:** 1 Department of Anesthesiology and Intensive Care, University of Rome, 'La Sapienza', Viale del Policlinico 155, Rome 00161, Italy; 2 Department of Neuroscience-Anesthesia and Intensive Care Unit, Università Politecnica delle Marche, Via Tronto 10, Torrette di Ancona 60020, Italy; 3 Department of Anesthesiology and Intensive Care, University Hospital of Muenster, Albert-Schweitzer-Str. 33, Muenster 48149, Germany; 4 Department of Anesthesia and Intensive Care, Università Vita-Salute San Raffaele, Via Olgettina 60, Milan 20132, Italy; 5 Hôpital de Bicêtre, Service of Medical Intensive Care, Centre Hospitalier de Bicêtre, rue du Général Leclerc 78, Le Kremlin-Bicêtre 94270, France; 6 Department of Translational Physiology, Academic Medical Center, University of Amsterdam, Meibergdreef 9, Amsterdam 1105 AZ, The Netherlands; 7 Department of Intensive Care, Erasmus MC, University Medical Center Rotterdam, 's-Gravendijkwal 230, Rotterdam 3015 CE, The Netherlands

## Abstract

**Introduction:**

The purpose of the present study was to investigate microcirculatory blood flow in patients with septic shock treated with levosimendan as compared to an active comparator drug (i.e. dobutamine). The primary end point was a difference of ≥ 20% in the microvascular flow index of small vessels (MFIs) among groups.

**Methods:**

The study was designed as a prospective, randomized, double-blind clinical trial and performed in a multidisciplinary intensive care unit. After achieving normovolemia and a mean arterial pressure of at least 65 mmHg, 40 septic shock patients were randomized to receive either levosimendan 0.2 μg·kg^-1^·min^-1 ^(*n *= 20) or an active comparator (dobutamine 5 μg·kg^-1^·min^-1^; control; *n *= 20) for 24 hours. Sublingual microcirculatory blood flow of small and medium vessels was assessed by sidestream dark-field imaging. Microcirculatory variables and data from right heart catheterization were obtained at baseline and 24 hours after randomization. Baseline and demographic data were compared by means of Mann-Whitney rank sum test or chi-square test, as appropriate. Microvascular and hemodynamic variables were analyzed using the Mann-Whitney rank sum test.

**Results:**

Microcirculatory flow indices of small and medium vessels increased over time and were significantly higher in the levosimendan group as compared to the control group (24 hrs: MFIm 3.0 (3.0; 3.0) vs. 2.9 (2.8; 3.0); *P *= .02; MFIs 2.9 (2.9; 3.0) vs. 2.7 (2.3; 2.8); *P *< .001). The relative increase of perfused vessel density vs. baseline was significantly higher in the levosimendan group than in the control group (dMFIm 10 (3; 23)% vs. 0 (-1; 9)%; *P *= .007; dMFIs 47 (26; 83)% vs. 10 (-3; 27); *P *< .001). In addition, the heterogeneity index decreased only in the levosimendan group (dHI -93 (-100; -84)% vs. 0 (-78; 57)%; *P *< .001). There was no statistically significant correlation between systemic and microcirculatory flow variables within each group (each *P *> .05).

**Conclusions:**

Compared to a standard dose of 5 μg·kg^-1^·min^-1 ^of dobutamine, levosimendan at 0.2 μg·kg^-1^·min^-1 ^improved sublingual microcirculatory blood flow in patients with septic shock, as reflected by changes in microcirculatory flow indices of small and medium vessels.

**Trial registration:**

NCT00800306.

## Introduction

Microvascular dysfunction plays a pivotal role in the pathophysiology of septic shock and may occur even in the presence of normal systemic oxygen supply and mean arterial pressure [1]. In this regard, several vasoactive agents, including inotropes, vasodilators, and inodilators, have been investigated in the attempt to preserve or improve microcirculatory blood flow in patients with severe sepsis or septic shock [1-5].

In recent years, much attention has been paid to the use of the calcium sensitizer levosimendan in the treatment of septic myocardial dysfunction [6-10]. Levosimendan increases myocardial contractility while simultaneously exerting vasodilatory properties via activation of ATP-dependent potassium channels (K_ATP_) [11]. In addition, levosimendan exerts anti-ischemic, anti-inflammatory, and anti-apoptotic properties, thereby affecting important pathways in the pathophysiology of septic shock [12-14]. It has been speculated that, owing to these beneficial effects, levosimendan may positively affect myocardial performance and regional hemodynamics, thereby improving microcirculatory perfusion [6-10,12,15,16].

The objective of the present randomized controlled, double-blinded clinical study was, therefore, to elucidate the effects of levosimendan on systemic and microvascular hemodynamics. On this basis, we aimed at rejecting the null hypothesis that there is no difference in sublingual microvascular blood flow - as measured by sidestream dark-field (SDF) imaging [17] - in patients with fluid-resuscitated septic shock treated with levosimendan as compared with an active comparator drug (that is, dobutamine).

## Materials and methods

### Patients

After approval by the local institutional ethics committee, the study was performed in an 18-bed multidisciplinary intensive care unit (ICU) at the Department of Anesthesiology and Intensive Care of the University of Rome 'La Sapienza'. Informed consent was obtained from the patients' next of kin. Enrolment of patients started in January 2008 and ended in April 2009. We enrolled patients who fulfilled the criteria of septic shock that required norepinephrine (NE) to maintain a mean arterial pressure (MAP) of at least 65 mm Hg despite appropriate volume resuscitation (pulmonary arterial occlusion pressure [PAOP] = 12 to 18 mm Hg and central venous pressure [CVP] = 8 to 12 mm Hg) [18]. Exclusion criteria of the study were age of less than 18 years, pregnancy, significant valvular heart disease, present or suspected acute coronary syndrome, and limitations to the use of inotropes (that is, ventricular outflow tract obstruction and mitral valve systolic anterior motion). All patients were sedated with sufentanil and midazolam and received mechanical ventilation using a volume-controlled mode.

### Hemodynamics, global oxygen transport, and acid-base balance

Systemic hemodynamic monitoring of the patients included a pulmonary artery catheter (7.5-F; Edwards Lifesciences, Irvine, CA, USA) and a radial artery catheter. MAP, right atrial pressure, mean pulmonary arterial pressure, and PAOP were measured at end-expiration. Heart rate was analyzed from a continuous recording of electrocardiogram with ST segments monitored. Cardiac index (CI) was measured using the continuous thermodilution technique (Vigilance II; Edwards Lifesciences). Systemic vascular resistance index, pulmonary vascular resistance index, and left and right ventricular stroke work indices were calculated by means of standard equations. Arterial and mixed-venous blood samples were withdrawn to determine oxygen tensions and saturations as well as carbon dioxide tensions, standard bicarbonate, base excess, pH, and lactate concentrations. SvO_2 _was measured discontinuously by intermittent mixed-venous blood gas analyses (Gem 4000 Premier; Instrumentation Laboratory Company, Bedford, MA, USA). Systemic oxygen delivery index (DO_2_I), oxygen consumption index, and oxygen extraction ratio were calculated by means of standard formulae.

### Microvascular network

Microvascular blood flow was visualized by means of an SDF imaging device (MicroScan^®^; MicroVision Medical, Amsterdam, The Netherlands) with a 5× magnification lens [17]. The optical probe was applied to the sublingual mucosa after gentle removal of saliva with a gauze swab. Three discrete fields were captured with precaution to minimize motion artifacts. Individual sequences of approximately 15 seconds were analyzed off-line with the aid of dedicated software (Automated Vascular Analysis 3.0; Academic Medical Center, University of Amsterdam, The Netherlands) in a randomized fashion by a single investigator who was unaware of the study protocol. Vessel density was automatically calculated from the software as the total vessel lengths of the small, medium, and large vessels, divided by the total area of the image [17]. The 'De Backer score' was calculated as described previously [17] and is based on the principle that density of the vessels is proportional to the number of vessels crossing arbitrary lines. In this score, three equidistant horizontal lines and three equidistant vertical lines are drawn on the screen, and then the De Backer score can be calculated as the number of small, medium, and large vessels crossing the lines, divided by the total length of the lines [17]. Vessel density was also calculated as the total vessel lengths divided by the total area of the image [17]. Both indices were automatically calculated by means of dedicated software (Automated Vascular Analysis 3.0). Perfusion was then categorized by eye as present (normal continuous flow for at least 15 seconds), sluggish (decreased but continuous flow for at least 15 seconds), absent (no flow for at least 50% of the time), or intermittent (no flow for less than 50% of the time) [17]. The proportion of perfused vessels (PPV) was calculated as follows: 100 × [(total number of vessels - [no flow + intermittent flow])/total number of vessels]. Perfused vessel density (PVD) was calculated by multiplying vessel density by the proportion of perfused vessels [17]. Microvascular flow index [17] was used to quantify microvascular blood flow. In this score, flow is characterized as absent (0), intermittent (1), sluggish (2), or normal (3) [17]. Since our investigation was focused on small and medium vessels, calculations were performed separately for vessels with diameters of smaller than 20 μm (MFIs) and of larger than 20 μm but smaller than 50 μm (MFIm). Vessel size was determined with the aid of a micrometer scale. For each patient, values obtained from the three mucosa fields were averaged [17]. To assess flow heterogeneity between the different areas investigated, we used the heterogeneity index. The latter was calculated as the highest site flow velocity minus the lowest site flow velocity, divided by the mean flow velocity of all sublingual sites [17]. Percentage changes from baseline for all variables were determined as dVariable = 100 × [(Value_24 hours _/Value_BL_) - 1] [19].

### Study design

**Figure 1:**
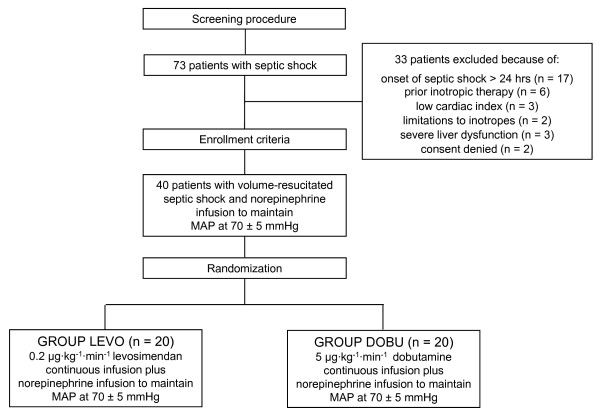
**Consort diagram**. MAP, mean arterial pressure.

Patients were enrolled within the first 24 hours from the onset of septic shock after having established normovolemia (PAOP = 12 to 18 mm Hg and CVP = 8 to 12 mm Hg) [18] and an MAP of at least 65 mm Hg using norepinephrine, if needed. Packed red blood cells were transfused when hemoglobin concentrations decreased to below 7 g/dL [18] or if the patient exhibited clinical signs of inadequate systemic oxygen supply. Forty patients were randomly allocated to the treatment with either (a) intravenous levosimendan 0.2 μg/kg per minute (without a loading bolus dose) for 24 hours or (b) intravenous dobutamine 5 μg/kg per minute as active comparator (= control) in a double-blinded manner (each *n *= 20). The consort diagram is presented in Figure 1. Systemic and pulmonary hemodynamic variables, microcirculatory flow variables, blood gases, and norepinephrine requirements were determined at baseline and 24 hours after randomization. After the 24-hour intervention period, study drugs were discontinued and open-label dobutamine was started if judged as appropriate by the attending ICU physician.

### Statistical analysis

An *a priori *analysis of sample size revealed that at least 17 patients per group were required to demonstrate a minimum difference of 20% between groups in the primary endpoint with an estimated standard deviation of 20%, a test power of 80%, and an alpha error of 5%. Data are expressed as median (25th; 75th percentile) if not otherwise specified. Sigma Stat 3.10 software (Systat Software, Inc., Chicago, IL, USA) was used for statistical analysis. Baseline and demographic data were compared with a Mann-Whitney rank sum test or chi-square test, as appropriate. Microvascular and hemodynamic variables were analyzed with a Mann-Whitney rank sum test. The correlation between systemic and microcirculatory flow variables within each group was tested by means of Spearman rank order correlation. A *P *value of less than 0.05 was considered statistically significant for all tests.

## Results

### Demographic data

Baseline characteristics, including age, gender, body weight, and origin, as well as onset time of septic shock, Simplified Acute Physiology Score II (SAPS II), and mortality were not different among groups (Table 1). In addition, there was no significant difference between groups at baseline in any of the investigated hemodynamic or microcirculatory variables.

**Table 1:** Characteristics of the study patients

	Levosimendan (*n *= 20)	Control (*n *= 20)	*P *value
Age, years	68 (55; 74)	66 (54; 78)	0.98
Gender, male	70%	65%	1.00
SAPS II	55 (45; 61)	57 (46; 64)	0.90
Cause of septic shock	Endocarditis (*n *= 1) Peritonitis (*n *= 8) Pneumonia (*n *= 11)	Peritonitis (*n *= 4) Pneumonia (*n *= 16)	0.10
Onset of septic shock, hours^a^	20 (18; 24)	18 (13; 22)	0.13
ICU mortality	13/20	15/20	0.50
ICU length of stay, days	14 (11; 19)	27 (9; 47)	0.32

Data are presented as median (25th; 75th percentile). Control, dobutamine 5 μg/kg per minute. ^a^Onset of septic shock defines the time elapsed from the onset of septic shock until administration of study drug. ICU, intensive care unit; SAPS II, Simplified Acute Physiology Score II.

### Hemodynamic and oxygen transport variables

Systemic and pulmonary hemodynamic variables were comparable between groups. SvO_2 _and arterial pH tended to be higher whereas NE requirements tended to be lower in the levosimendan group (Table 2). However, these differences did not reach statistical significance.

**Table 2:** Hemodynamic and metabolic data of the study patients

		Levosimendan (*n *= 20)	Control (*n *= 20)	*P *value
CI, L/min per m^2^	BL	3.6 (2.9; 4.3)	3.9 (2.9; 4.6)	0.70
	24 hours	4.1 (3.5; 5.1)^a^	4.1 (3.3; 5.0)	0.66
HR, beats per minute	BL	96 (87; 107)	95 (90; 106)	0.75
	24 hours	94 (86; 104)	98 (87; 114)	0.36
MAP, mm Hg	BL	70 (67; 72)	72 (70; 74)	0.11
	24 hours	72 (69; 73)	73 (70; 75)	0.13
PAOP, mm Hg	BL	18 (15; 18)	19 (15; 21)	0.25
	24 hours	16 (16; 18)	17 (14; 21)	0.52
RAP, mm Hg	BL	14 (11; 16)	14 (11; 16)	0.81
	24 hours	13 (11; 14)	14 (10; 18)	0.27
LVSWI, g·m/m^2^	BL	26 (21; 32)	30 (25; 36)	0.13
	24 hours	34 (29; 38)^a^	32 (29; 38)	0.56
DO_2_I, mL/min per m^2^	BL	431 (363; 531)	492 (393; 550)	0.27
	24 hours	512 (438; 612)	519 (436; 593)	0.93
VO_2_I, mL/min per m^2^	BL	111 (93; 151)	126 (112; 153)	0.18
	24 hours	127 (107; 144)	149 (110; 178)	0.24
O_2_-ER, percentage	BL	28 (24; 32)	29 (22; 34)	0.99
	24 hours	25 (20; 27)^a^	27 (21; 36)	0.17
SaO_2_, percentage	BL	98 (96; 99)	98 (95; 99)	0.99
	24 hours	99 (99; 99)^a^	99 (94; 99)	0.02
PaCO_2_, mm Hg	BL	45 (41; 50)	41 (37; 51)	0.35
	24 hours	41 (37; 44)	41 (36; 49)	0.42
SvO_2_, percentage	BL	72 (66; 75)	70 (66; 78)	0.95
	24 hours	77 (74; 81)^a^	71 (62; 78)	0.06
Hb_a_, g/dL	BL	8.6 (8.0; 8.9)	9.0 (8.0; 9.6)	0.96
	24 hours	8.5 (8.0; 8.9)	8.8 (8.0; 9.3)	0.42
pH_a_, -log_10_c(H^+^)	BL	7.29 (7.25; 7.34)	7.28 (7.25; 7.38)	0.87
	24 hours	7.38 (7.29; 7.40)^a^	7.32 (7.23; 7.37)	0.06
aBE, mmol/L	BL	-4.9 (-6.9; -2.5)	-3.8 (-9.0; 0.0)	0.72
	24 hours	-2.9 (-5.0; -0.6)	-3.8 (-8.9; 1.8)	0.74
Lactate, mmol/L	BL	2.3 (1.3; 2.9)	1.9 (1.3; 2.9)	0.72
	24 hours	1.9 (1.2; 2.5)	1.6 (1.3; 3.6)	0.61
Fluid input, mL/24 hours	BL	NA	NA	NA
	24 hours	5,700 (4,700; 6,050)	4,850 (4,150; 5,200)	0.01
NE dosage, μg/kg per min	BL	0.4 (0.2; 0.9)	0.4 (0.3; 0.7)	0.72
	24 hours	0.3 (0.1; 0.9)	0.4 (0.3; 1.1)	0.10

Data are presented as median (25th; 75th percentile). Control, dobutamine 5 μg/kg per minute. ^a^*P *< 0.05 versus baseline (BL) within groups. aBE, arterial base excess; CI, cardiac index; DO_2_I, systemic oxygen delivery index; Hb_a_, arterial hemoglobin concentration; HR, heart rate; LVSWI, left ventricular stroke work index; MAP, mean arterial pressure; NA, not applicable; NE, norepinephrine; O_2_-ER, oxygen extraction ratio; PaCO_2_, arterial partial pressure of carbon dioxide; PAOP, pulmonary arterial occlusion pressure; pH_a_, arterial *potentia hydrogenii*; RAP, right atrial pressure; SaO_2_, arterial oxygen saturation; SvO_2_, mixed-venous oxygen saturation; VO_2_I, oxygen consumption index.

### Concomitant therapies

Activated protein C was administered in five patients in the control group and in four patients in the levosimendan group. Three patients in each group required continuous renal replacement therapy during the study period. These treatments were equally distributed among groups (each *P *value of greater than 0.05).

### Microcirculatory variables

**Figure 2:**
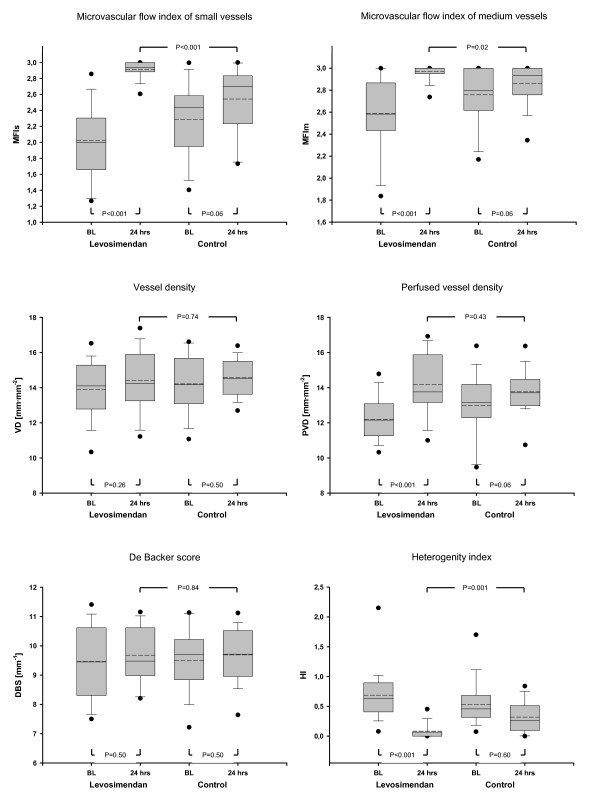
**Absolute changes in microcirculatory variables**. BL, baseline; DBS, De Backer score; HI, heterogenity index; MFIm, microvascular flow index of medium vessels (∅ 20 to 50 μm); MFIs, microvascular flow index of small vessels (∅ <20 μm); PVD, perfused vessel density; VD, vessel density.

**Figure 3:**
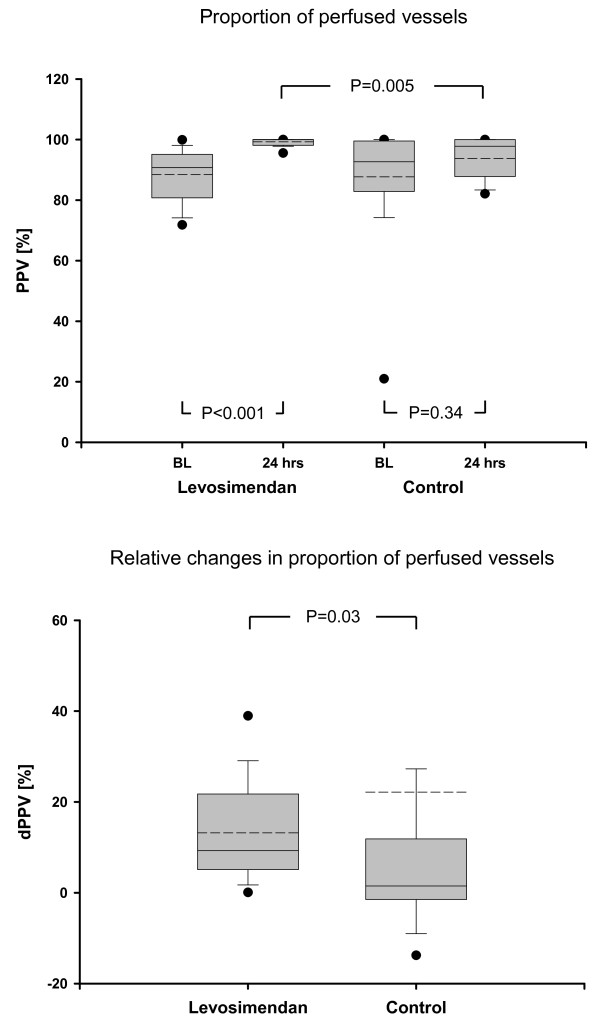
**Absolute and relative changes in microcirculatory variables**. BL, baseline; dPPV, relative changes in proportion of perfused vessels; PPV, proportion of perfused vessels.

**Figure 4:**
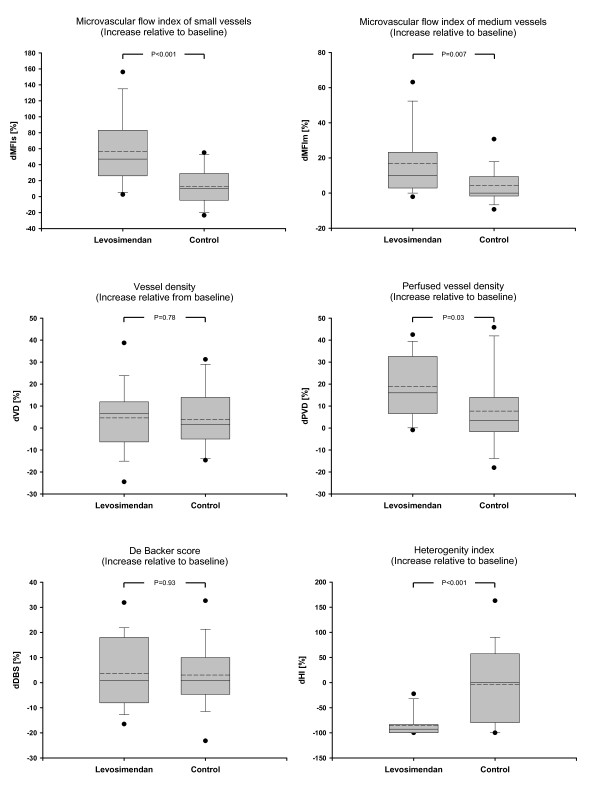
**Relative changes in microcirculatory variables**. Data represent relative changes from baseline at 24 hours. dDBS, relative changes in De Backer score; dHI, relative changes in heterogeneity index; dMFIm, relative changes in microvascular flow index of medium vessels (∅ 20 to 50 μm); dMFIs, relative changes in microvascular flow index of small vessels (∅ <20 μm); dPVD, relative changes in perfused vessel density; dVD, relative changes in vessel density.

**Figure 5:**
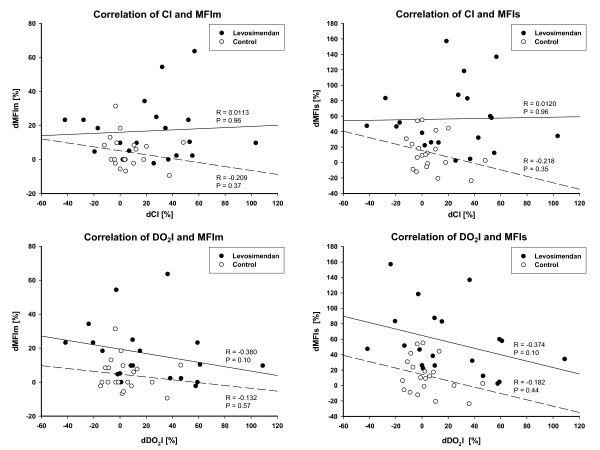
**Correlation analyses of systemic and microcirculatory flow variables**. Data represent percentage changes in cardiac index (dCI) and systemic oxygen delivery index (dDO_2_I) plotted against percentage changes in microvascular flow indices of medium (dMFIm) and small (dMFIs) vessels within each group. Solid and dashed lines represent regression lines for levosimendan and control, respectively. CI, cardiac index; DO_2_I, systemic oxygen delivery index; MFIm, microvascular flow index of medium vessels (∅ 20 to 50 μm); MFIs, microvascular flow index of small vessels (∅ <20 μm).

Microcirculatory data are presented in Figures 2, 3 and 4. MFIm and MFIs were significantly higher (MFIm 3.0 [3.0; 3.0] versus 2.9 [2.8; 3.0]; *P *= 0.02; MFIs 2.9 [2.9; 3.0] versus 2.7 [2.3; 2.8]; *P *< 0.001) and heterogenity index was lower after 24 hours of treatment with levosimendan versus dobutamine (heterogenity index 0.63 [0.44; 0.87] versus 0.26 [0.12; 0.51]; *P *= 0.001). Since baseline data varied (non-significantly) among groups, relative changes from baseline were calculated and compared between groups. Relative increases from baseline of MFIs, MFIm, PPV, and PVD (that is, dMFIs, dMFIm, dPPV, and dPVD) were significantly higher in the levosimendan group (Figure 3 and 4). In addition, the heterogeneity index decreased relative to baseline only in the levosimendan group. Correlation analyses (that is, DO_2_I and CI versus MFIm and MFIs in each group) revealed no statistically significant results (each *P *> 0.05; Figure 5).

## Discussion

The major finding of the present study is that levosimendan improved microvascular perfusion in patients with septic shock, as indicated by increases in MFIs, MFIm, and PVD. Notably, this improvement was related to enhanced convection rather than changes in diffusion distance.

The role of levosimendan in severe sepsis or septic shock is still not fully elucidated and remains controversial [12,14-16,20-26]. However, there is increasing evidence that under normovolemic conditions, continuous infusion with levosimendan attenuates septic myocardial dysfunction [6-10,27,28] without aggravating hemodynamic instability. In harmony with previous reports [6-10,27,28], levosimendan did not influence arterial blood pressure or NE requirements in the present study. Furthermore, we noticed neither an increase in heart rate nor new onsets of tachyarrhythmias following levosimendan infusion in our fluid-resuscitated septic shock patients. These findings strengthen the assumption that under normovolemic conditions, the decrease in vascular resistance (owing to the opening of K_ATP _channels) following levosimendan infusion may be compensated by a simultaneous increase in myocardial contractility.

The hypothesis that constituted the basis of our study was that (besides the effects on myocardial contractility) levosimendan - by its vasodilatory effects - improves microcirculatory blood flow by increasing the driving pressure of blood flow at the entrance of the microcirculation [3]. In fact, we noticed that levosimendan improved sublingual microcirculation, as indicated by significant increases in MFIs, MFIm, dMFIs, and dMFIm. In addition, we observed an increase in dPVD following levosimendan infusion, further indicating an improvement of the microcirculation. We focused our investigation on the effects of the study drug on MFI of the small and medium vessels since alterations in such microvessels are typically associated with organ dysfunction and - if persisting - poor outcome [1-5].

Whereas the increases in MFI suggest that levosimendan ameliorated blood flow within the perfused vessels, the increase in PPV with a concomitant decrease in heterogeneity index indicates a recruitment of non-perfused vessels and hence a reduction of the diffusion distance between capillaries. In light of these findings, it is most likely that levosimendan enhanced both convection and diffusion, thereby improving oxygen delivery at the level of the microcirculation.

Although the increases in SvO_2 _and pH noticed in the levosimendan group may further indicate an improvement in microcirculatory blood flow, it has to be considered that an improvement in pulmonary function (increase in PaO_2 _[arterial oxygen partial pressure] and SaO_2 _[arterial oxygen saturation] with a concomitant decrease in PaCO_2 _[arterial partial pressure of carbon dioxide]) following levosimendan administration might have contributed to these changes. This assumption is supported by recent experimental and clinical studies showing that levosimendan in fact improves pulmonary function and gas exchange [8,12,14,20,25,26]. However, it may well be that levosimendan (secondary to its vasodilatatory properties) has promoted microvascular shunting and thereby increased venous oxygen saturation.

Our results are in line with those of an experimental study by Schwarte and colleagues [29], who reported that levosimendan selectively increases gastric microvascular mucosal oxygenation in dogs. Whereas a previous experimental study [30] showed that levosimendan improved microvascular oxygenation in experimental sepsis, our study demonstrates for the first time that levosimendan selectively increases microvascular blood flow in the clinical setting. However, the present study design does not allow us to exclude whether non-hemodynamic effects of levosimendan, such as the ability to decrease cytokine synthesis, plasma levels of endothelin-1, ICAM-1 (intercellular adhesion molecule-1), and VCAM-1 (vascular cell adhesion molecule-1) [12,13,26], might have contributed to the improvement of microcirculation.

Notably, the lack of modifications in the proportion of perfused vessels observed in the control group (in which the patients were treated with dobutamine as an active comparator at a dose of 5 μg/kg per minute) varies from the study of De Backer and colleagues [2], who reported that the same dose of dobutamine increased microvascular density and the proportion of perfused vessels, a finding that clearly indicated an improved microcirculation in a series of septic shock patients. However, despite the use of an equivalent dobutamine dose [2], there is a marked difference in the study designs in terms of time frame. In this regard, the previously reported short-term response to dobutamine after 2 hours [2] was outside the scope of our investigation. A likely explanation might be related to the fact that we performed microcirculatory evaluation at the end of 24 hours of drug infusion in progressed septic shock. It is well recognized that, owing to adrenergic receptor and signaling abnormalities, the efficacy of catecholamines often gradually decreases over time [31]. This may account for the attenuated hemodynamic effects of 5 μg/kg per minute dobutamine infusion in patients with severe septic shock [7,32,33] in comparison with patients with less severe sepsis [34]. On this basis, it is conceivable that microvessels may reach a near maximal vasodilation in the early phase of dobutamine administration lasting for a brief period [2,32,35], whereas after 24 hours, the effects of 5 μg/kg per minute of dobutamine on the microcirculation are attenuated. In this light, our findings support the hypothesis formulated by De Backer and colleagues [2] that stronger vasodilatory compounds, such as levosimendan, may be more effective than dobutamine for improving microcirculatory blood flow. However, these postulated advantages of levosimendan remain to be further elucidated in larger clinical trials.

The present study has some limitations that we would like to acknowledge. First, we administered a fixed dose of 5 μg/kg per minute of dobutamine and cannot exclude the possibility that a higher dose would have resulted in different findings. However, it is important to note that our intention was not to perform a direct comparison between dobutamine and levosimendan but to use the selected dobutamine dose as an 'active comparator' to facilitate blinding of the study drugs. Indeed, randomization of levosimendan versus placebo would have unmasked group allocation because of the strong hemodynamic effects of levosimendan. Second, in the present study, the improvement in microvascular perfusion was independent from changes in CI. However, it is also possible that these variables might correlate in a way that is more complex than the linear correlation of percentage changes in CI and oxygen delivery. Therefore, a possible correlation should be clarified in future larger studies. Third, owing to the lack of investigation of specific variables, we cannot conclude whether anti-ischemic and anti-inflammatory effects, as well as effects at the cellular level [13], have contributed to the improved microcirculatory blood flow with levosimendan. In addition, we investigated the changes in microvascular perfusion of the sublingual mucosa which might not be representative of alterations in other tissues [1]. Furthermore, owing to the pharmacokinetic characteristics of the study drug, the present study protocol required a relatively long time interval (24 hours of drug infusion) that does not allow the exclusion of a direct time-dependent effect unrelated to the specific agent. Finally, we have chosen changes in MFIs as the primary endpoint of this study. Since we investigated only a small number of septic shock patients treated over a relative brief period, the risk of positive results in a study with numerous secondary variables has to be taken into account. Thus, caution should be exercised in interpreting the results of the secondary outcome variables.

## Conclusions

This is the first prospective, randomized clinical study investigating the effects of levosimendan on sublingual microcirculation in patients with septic shock. Our results demonstrate that levosimendan at 0.2 μg/kg per minute (when compared with a standard dose of 5 μg/kg per minute of dobutamine) improves sublingual microcirculatory blood flow in volume-resuscitated septic shock patients and that this effect was not correlated with changes in systemic flow variables.

## Key messages

• Levosimendan improves sublingual microcirculatory blood flow in volume-resuscitated septic shock patients.

• Levosimendan enhances convection and improves diffusion, thereby improving oxygen delivery at the level of the microcirculation.

• Levosimendan at 0.2 μg/kg per minute may be more effective than a standard dose of 5 μg/kg per minute of dobutamine for improving microcirculatory blood flow.

• Under normovolemic conditions, levosimendan administration did not influence arterial blood pressure or norepinephrine requirements.

## Abbreviations

CI: cardiac index; CVP: central venous pressure; dMFIm: relative increases of microvascular flow index of medium vessels; dMFIs: relative increases of microvascular flow index of small vessels; DO_2_I: systemic oxygen delivery index; dPVD: relative increase in perfused vessel density; ICU: intensive care unit; K_ATP_: ATP-dependent potassium; MAP: mean arterial pressure; MFIm: microvascular flow index of medium vessels; MFIs: microvascular flow index of small vessels; NE: norepinephrine; PAOP: pulmonary arterial occlusion pressure; PPV: proportion of perfused vessels; PVD: perfused vessel density; SDF: sidestream dark-field; SvO_2_: mixed-venous oxygen saturation.

## Competing interests

The authors declare that they have no competing interests.

## Authors' contributions

AM and MW planned the study, were responsible for its design and coordination, and drafted the manuscript. J-LT and GL participated in the study design and helped to draft the manuscript. CE, ML, SR, and HVA participated in the design of the study, performed the statistical analysis, and helped to draft the manuscript. AO, VC, AD, P Pelaia, and CI analyzed SDF images and helped to draft the manuscript. P Pietropaoli participated in the study design, helped to draft the manuscript, and obtained funding. All authors read and approved the final manuscript.
